# Low prevalence of concurrent active hepatitis E and B virus infection in high-risk groups in Southwestern Cameroon

**DOI:** 10.1016/j.ijregi.2025.100745

**Published:** 2025-09-05

**Authors:** Macqueen Ngum Mbencho, Eric A. Achidi, Stephen Mbigha Ghogomu, Thirumalaisamy P. Velavan

**Affiliations:** 1 Institute of Tropical Medicine, University of Tübingen and German Center for Infection Research (DZIF), Tübingen, Germany; 2 Molecular and Cell Biology Laboratory, University of Buea, Buea, Cameroon; 3 Faculty of Sciences, University of Buea, Buea, Cameroon; 4 Vietnamese German Center for Medical Research (VG-CARE), Hanoi, Vietnam; 5 Faculty of Medicine, Duy Tan University, Da Nang, Vietnam

**Keywords:** Hepatitis E, Hepatitis B, HIV, Coinfection, Cameroon

## Abstract

**Objectives:**

Coinfection with the hepatitis E virus (HEV) and hepatitis B virus (HBV) can have clinical implications, particularly in immunocompromised and high-risk populations. This study investigated HEV seroprevalence and viraemia among individuals with HBV exposure in the Southwest region of Cameroon.

**Methods:**

We analyzed three cohorts with prior HBV exposure, defined as anti-HBc total positivity: HIV-infected individuals (n = 174), pregnant women (n = 87), and blood donors (n = 167). Participants were screened for anti-HEV immunoglobulin (Ig)G and IgM using an enzyme-linked immunosorbent assay, and HEV RNA was detected by reverse transcription-polymerase chain reaction. HBV DNA was quantified in those with hepatitis B surface antigen positivity.

**Results:**

Among anti-hepatitis B core total-positive participants, anti-HEV IgG seroprevalence was 8.6% in HIV-infected individuals, 5.7% in pregnant women, and 12.0% in blood donors. Anti-HEV IgM was detected in 1.7%, 0%, and 1.2%, respectively. Also, HEV RNA was detected in two blood donors (genotype 3a) and two pregnant women (genotype 3e), but not in HIV-infected individuals. No cases of concurrent active HBV and HEV replication were observed.

**Conclusions:**

Previous HEV exposure is relatively common in this region, but active HBV-HEV coinfection appears rare among HIV-infected individuals, pregnant women, and blood donors in Southwest Cameroon.

## Highlights

• Hepatitis E virus (HEV) exposure is common in Southwest Cameroon.
• Study covers HIV patients, pregnant women, and blood donors.
• No cases of simultaneous hepatitis B virus and HEV replication detected.
• Findings support targeted HEV surveillance in high-risk groups.

## Introduction

Hepatitis E virus (HEV) is an emerging cause of acute viral hepatitis worldwide, with the highest burden in low- and middle-income countries, particularly in Africa and Asia [1]. The African region is also disproportionately affected by chronic hepatitis B virus (HBV) infection, driven largely by perinatal transmission and sustained high co-endemicity with HIV [2]. Coinfection with HEV and HBV can exacerbate liver damage, especially in immunocompromised individuals such as HIV-infected individuals and pregnant women [3,4]. In settings where HEV and HBV are co-circulating, dual infection can result in acute-on-chronic liver failure and increased mortality, especially among those with HBV-related cirrhosis [5]. Despite the potential clinical severity, the prevalence and clinical significance of concurrent HEV and HBV infection in different population groups remain insufficiently characterized in Central Africa. This study aimed to determine the prevalence of past HEV exposure, recent infection, and active HEV viremia in three distinct population cohorts from southwestern Cameroon, and to assess their co-occurrence with markers of HBV replication.

## Methods

### Ethical approval

The study was approved by the Institutional Review Board of the University of Buea, Cameroon (Reference: 2022/1849-10/UB/SG/IRB/FHS), and by the Ethics Committee of the University of Tübingen, Germany (Reference: 379/2023B02). Administrative clearance was obtained from the Southwest Regional Delegation of Public Health. Written informed consent was obtained from all participants before enrollment.

### Laboratory investigations

We performed serological and molecular screening for HEV and HBV markers in three cohorts recruited in the Southwest region of Cameroon that included HIV-infected individuals (n = 233), pregnant women (n = 190), and blood donors (n = 289). Anti-hepatitis B core (anti-HBc) total, HBV deoxyribonucleic acid (DNA), and HBV viral load were assessed to determine HBV status. HEV exposure and infection were evaluated using anti-HEV immunoglobulin (Ig)G, anti-HEV IgM, and HEV ribonucleic acid (RNA) detection by reverse transcription-polymerase chain reaction, as described in our previous studies [6,7].

## Results

Among participants who tested positive for anti-HBc total, anti-HEV IgG was detected in 8.6% (15/174) of HIV-infected individuals, 5.7% (5/87) of pregnant women, and in 12% (20/167) of blood donors (Table 1). Anti-HEV IgM positivity was rare and was observed in 1.7% (3/174) of HIV-infected individuals, none among the pregnant women, and 1.2% (2/167) of blood donors (Table 1). HEV RNA positivity was detected in only two blood donors (genotype 3a) and two pregnant women (genotype 3e), while no cases were detected among HIV-infected participants. Importantly, no cases of HEV RNA positivity occurred in individuals with detectable HBV DNA. Among HBV DNA-positive individuals—24 (10%) in the HIV cohort, 7 (3%) in the pregnant women cohort, and 14 (5%) in the blood donor cohort—none had detectable anti-HEV IgM or HEV RNA (Table 1), suggesting the absence of concurrent active HBV and HEV replication in the study population. Furthermore, among anti-HBc total-positive individuals across cohorts, markers of recent or active HEV infection (anti-HEV IgM and/or HEV RNA) were more common in the 21–40 years age group, whereas previous HEV exposure (anti-HEV IgG) was predominant in older adults above 40 years.

**Table 1:** Prevalence of HEV exposure, recent infection, and active viremia in relation to HBV markers across study cohorts in Southwestern Cameroon.

Study cohort	HBV parameters	Anti-HEV IgG positivity	Anti-HEV IgM positivity	HEV-RNA Positivity
HIV patients	Anti-HBc total positivity (n = 174)	15 (8.6%)	3 (1.7%)	0
	HBV-DNA positivity (n = 24)	1 (4.2%)	0	0
Pregnant women	Anti-HBc total positivity (n = 87)	5 (5.7%)	0	2 (2.3%)
	HBV-DNA positivity (n = 7)	0	0	0
Blood donors	Anti-HBc total positivity (n = 167)	20 (12%)	2 (1.2%)	2 (1.2%)
	HBV-DNA positivity (n = 14)	0	0	0

HBc, hepatitis B core; HBV, hepatitis B virus; HEV, hepatitis E virus; Ig, immunoglobulin.

## Discussion

In this HBV-endemic setting, previous HEV exposure was detected in all three cohorts, with the highest prevalence among blood donors, intermediate among HIV-infected individuals, and lowest among pregnant women.

While there is substantial evidence for exacerbated liver disease with HEV coinfection or superinfection in various populations with different underlying HBV-related liver disease stages [8], reports for those living with HIV are scarce. The absence of concurrent active HBV-HEV replication in the HIV cohort is consistent with reports from other endemic regions [8,9], potentially explained by the transient nature of HEV viremia, non-overlapping transmission routes, and possible immune-mediated viral interference [8,10]. The relatively low anti-HEV IgG prevalence in HIV-infected individuals compared to similar settings may relate to differences in exposure patterns or assay sensitivity, as importantly, no active HEV infection was observed in this group despite their higher risk of chronicity. Given the heightened risk of HIV acquisition associated with HBV in endemic regions and the potential for accelerated liver disease progression in coinfected individuals, our findings underscore the need for targeted public health strategies to prevent HEV in this and similar settings.

The age distribution of HEV markers, with recent infection in younger adults and past exposure in older adults, matches the known epidemiology in endemic regions [1], while HBV infection in this setting is typically acquired in early infancy [2]. Our data indicate a low prevalence of recent or active HEV infection among pregnant women. Studies from similar settings have reported higher HEV seropositivity in HBV-positive pregnant women compared to HBV-negative, with coinfection associated with increased obstetric complications and adverse birth outcomes [4]. HEV RNA was detected in two pregnant women of genotype 3e, with negative anti-HEV IgM, suggesting early infection with a low-titer IgM response. No severe courses were observed during the study, nor presumed, as severe outcomes have predominantly been associated with genotype 1 [4,7]. Nevertheless, close monitoring remains essential to mitigate any potential risks for both mother and child.

In HBV-endemic regions like Cameroon, with a high prevalence of anti-HBc total in the general population [6], the high rate of HEV exposure among blood donors likely reflects a similar level of community-wide exposure to HEV. This pattern suggests that HEV transmission and exposure may be driven by factors such as water contamination, poor sanitation, and food-borne exposure that affect the wider population. Notably, in our study, no blood donor exhibited concurrent HBV and HEV viraemia. This absence of dual active replication aligns with observations from other endemic settings, where co-exposure is relatively common but simultaneous replication of both viruses is rare [8,9].

Taken together, in a region with high HBV exposure, HEV co-exposure is relatively common, but active infection is rare, and simultaneous replication of both viruses was not detected. These results underscore the need for continuous HEV surveillance, particularly in pregnant women and immunocompromised individuals, and support the integration of HEV education and prevention into existing HBV and HIV programs. Further longitudinal studies are needed to investigate the temporal dynamics, risk factors, and clinical consequences of HEV-HBV coinfection in this and similar settings.

## Declaration of competing interest

The authors have no competing interests to declare.
